# Application of Whole Exome and Targeted Panel Sequencing in the Clinical Molecular Diagnosis of 319 Chinese Families with Inherited Retinal Dystrophy and Comparison Study

**DOI:** 10.3390/genes9070360

**Published:** 2018-07-19

**Authors:** Likun Wang, Jinlu Zhang, Ningning Chen, Lei Wang, Fengsheng Zhang, Zhizhong Ma, Genlin Li, Liping Yang

**Affiliations:** 1 Institute of Systems Biomedicine & Department of Ophthalmology, School of Basic Medical Sciences, Third Hospital, Peking University, Beijing 100191, China; wanglk@bjmu.edu.cn (L.W.); zhangjinlu@bjmu.edu.cn (J.Z.); ningningchen12@163.com (N.C.); puh3_yk@bjmu.edu.cn (Z.M.); 2 Beijing Key Laboratory of Tumor Systems Biology, Department of Pathology, School of Basic Medical Sciences, Peking University Health Science Center, Beijing 100191, China; 3 Peking University Third Hospital, Beijing Key Laboratory of Restoration of Damaged Ocular Nerve, Beijing 100191, China; 4 Beijing Tongren Eye Center, Beijing Tongren Hospital, Capital Medical University, Beijing Ophthalmology & Visual Sciences Key Lab, Beijing 100730, China; wl998295@163.com; 5 Huhehaote Chaoju Eye Hospital, No. 40, W. Railway Station Road New City District, Huhehaote 010050, China; zhangfengsheng@chaojueye.cn

**Keywords:** inherited retinal dystrophy, whole exome sequencing, targeted panel sequencing, molecular diagnosis

## Abstract

Inherited retinal dystrophies (IRDs) are a group of clinically and genetically heterogeneous diseases involving more than 280 genes and no less than 20 different clinical phenotypes. In this study, our aims were to identify the disease-causing gene variants of 319 Chinese patients with IRD, and compare the pros and cons of targeted panel sequencing and whole exome sequencing (WES). Patients were assigned for analysis with a hereditary eye disease enrichment panel (HEDEP) or WES examination based on time of recruitment. This HEDEP was able to capture 441 hereditary eye disease genes, which included 291 genes related to IRD. As *RPGR* ORF15 was difficult to capture, all samples were subjected to Sanger sequencing for this region. Among the 163 disease-causing variants identified in this study, 73 had been previously reported, and the other 90 were novel. Genes most commonly implicated in different inheritances of IRDs in this cohort were presented. HEDEP and WES achieved diagnostic yield with 41.2% and 33.0%, respectively. In addition, nine patients were found to carry pathogenic mutations in the *RPGR* ORF15 region with Sanger sequencing. Our study demonstrates that HEDEP can be used as a first-tier test for patients with IRDs.

## 1. Introduction

Inherited retinal dystrophies (IRDs) are a group of clinically and genetically heterogeneous diseases characterized by progressive degeneration of photoreceptors and/or the retinal pigment epithelial cells. Collectively, IRDs have an incidence rate ranging from 1 in 2000 to 1 in 3000, and affect almost two million people worldwide [1]. In most cases, the retina is the only affected tissue (non-syndromic forms). However, in some patients, other tissues may also be involved (syndromic form) [2], and these syndromic forms include Usher syndrome, Bardet–Biedl syndrome, Senior–Loken syndrome and so on. To date, more than 280 genes have been shown to be associated with IRD, and all modes of genetic inheritance are involved. This clinical and genetic heterogeneity hamper the efficiency and dependability of clinical molecular diagnosis of IRD.

Next-generation sequencing (NGS) is a powerful technique that enables rapid and cost-effective parallel sequencing of large panels of genes. The targeted panel sequencing approach, which has been shown to provide deep coverage of the targeted sequences, has been widely used in the molecular diagnosis of IRD [3,4,5]. At the same time, accompanying the marked increase in sequencing content and dramatic decrease per base sequencing cost, the use of whole exome sequencing (WES) in IRD has also increased significantly [6,7]. As both WES and targeted panel sequencing yield accurate genetic diagnoses, clinicians are faced with the challenge of deciding which method to use.

In this study, we describe a cohort of 319 individuals affected by IRD. In this cohort, 228 patients had a specific hereditary eye disease enrichment panel (HEDEP) examination, while the other 91 patients had a WES examination. Our aim was to identify the disease-causing variants in this large cohort, and to provide useful information for clinicians to make diagnosis by comparing the data quality and diagnostic yield of targeted panel sequencing and WES.

## 2. Materials and Methods

### 2.1. Ethics Statement

This study conformed to the tenets of the Declaration of Helsinki. All experiments involving patient DNA and DNA of patient relatives were approved by the Peking University Third Hospital Medical Ethics Committee (No. 2012093). Written informed consents were obtained from all participants, or from guardians on behalf of minors/child participants. The ethics committee approved this consent procedure.

### 2.2. Patients

A cohort of 319 Chinese families of Han ethnicity with IRD was recruited from the Department of Ophthalmology, Peking University Third Hospital, and Beijing Tongren Eye Center. A detailed family history was obtained from the probands and/or their relatives. All patients underwent standard ophthalmic examinations including best-corrected visual acuity (BCVA), slit-lamp biomicroscopy, dilated indirect ophthalmoscopy, fundus photography, fluorescent angiography (FFA), optical coherence tomography (OCT), electroretinograms (ERG), and visual field tests if possible. The ERG protocol complied with the standards published by the International Society for Clinical Electrophysiology of Vision. Diagnostic evaluation for IRD was performed by at least two retinal specialists, systemic examination was performed before genetic analysis. The clinical diagnoses of participating subjects included retinitis pigmentosa (RP), Leber congenital amaurosis (LCA), cone-rod dystrophy (CORD), Stargardt disease, congenital stationary night blindness (CSNB), Usher syndrome, Bardet–Biedl syndrome, Best disease, macular dystrophy and so on. One hundred healthy individuals from the Chinese Han ethnic population were recruited as controls.

### 2.3. Design of Targeted Capture Panel

A specific HEDEP based on exon capture technology was used to collect the protein coding regions of targeted genes. This HEDEP was able to capture 441 hereditary eye disease genes, which included 291 genes related to IRD. The other genes were related to corneal dystrophy, congenital cataract, open angle glaucoma and so on. The panel was custom designed and produced (Agilent, Santa Clara, CA, USA) according to the manufacturer’s instructions. A total of 228 IRD probands were subjected to HEDEP for targeted exon capture, and exon-enriched DNA libraries were then prepared for high throughput sequencing with the Illumina HiSeq X platform (Illumina, San Diego, CA, USA) using 150 bp paired-end reads.

### 2.4. Whole Exome Sequencing

Whole exome sequencing was performed using an Agilent’s SureSelect Human All Exon V6 Kit (Agilent, Santa Clara, CA, USA) in conjunction with the Illumina HiSeq X platform (Illumina, San Diego, CA, USA) using 150 bp paired-end reads. The WES kit was able to provide high coverage of exonic regions (up to 60M bp). A total of 91 IRD probands were subjected to WES. The selection criterion for patients who were subjected to WES was based on time of recruitment only (individuals recruited between 21 September and 24 October 2016), and no other standards were considered.

### 2.5. Next-Generation Sequencing Data Analysis

HEDEP and WES sequencing data were analyzed as described previously [8]. In brief, sequencing alignments were performed using the Burrows-Wheeler Alignment (BWA) tool [9]. After read mapping, GATK [10] was used to realign the raw gapped alignments in order to reduce the number of miscalls from short insertions or deletions (indel). During data processing, GATK was also used to reduce the effects of analysis artifacts produced during sequencing by adjusting the quality scores for each read. GATK together with Samtools [11] was used for variant calling to identify single nucleotide polymorphisms (SNPs) and indels. CoNIFER [12] was used to find disruptive gene copy number variants(CNVs). ANNOVAR [13] was performed to annotate the SNPs and indels. All candidate mutations were filtered against the ExAC [14] project to remove polymorphic loci. Genomic evolutionary rate profiling (GERP) scores were calculated using the GERP++ package [15]. PolyPhen-2 [16], SIFT [17], and Mutation Taster [18] were used for functional predictions. ExAC exome allele frequency data were primarily used to estimate the minor allele frequency (MAF) and remove polymorphic loci. The threshold was set as 0.01 or 0.0001 for a potentially novel recessive gene, or dominant disease-causing variants respectively.

### 2.6. Variant Classification and Validation

Sequence changes were classified according to the American College of Medical Genetics and Genomics and the Association for Molecular Pathology (ACMG/AMP) variant interpretation guidelines [19,20]. In this study, only those variants identified as pathogenic (frameshift or premature translational termination occurring before the penultimate exon, essential splice site variants, and reported variants that are known to cause retinal diseases in the HGMD [21] professional database) or likely pathogenic (novel missense variants that are predicted to be pathogenic by in silico prediction algorithms such as PolyPhen-2 [16], SIFT [17], and Mutation Taster [18] for functional prediction, as well as those affecting a conserved residue, and when two mutated alleles of the same gene were identified that were inherited from their parents separately) were reported. Those variants of uncertain significance (if only one variant was identified in a presumed autosomal recessive patient), likely benign or benign were not included. All variants considered to be disease-causing were confirmed with Sanger sequencing, and co-segregation analyses were performed in available family members.

### 2.7. Regions of Low Coverage with Next Generation Sequencing

Mutations in *RPGR* gene account for a substantial proportion of patients with RP, and most mutations are detected in ORF15 [22]. Based on our coverage analysis for each base pair, the *RPGR* exon ORF15 was not effectively covered by NGS, and it was therefore PCR amplified and Sanger sequenced. Amplification of the *RPGR* exon ORF15 (NG_009553.1; NM_001034853) was carried out using primers located outside the repetitive stretch as described previously [22]. The forward primer was 5${}^{\prime }$-CAGAGATCCTATCAGATGACC-3${}^{\prime }$, and the reverse primer was 5${}^{\prime }$-TGTCTGACTGGCCATAATCG-3${}^{\prime }$, with an expected PCR product of 1630 bp. PCR products were sequenced using four previously reported reverse primers [22]. Sequencing results were analyzed with Sequencher (Gene Codes Corporation, Ann Arbor, MI, USA). The mutations which were identified were further evaluated for segregation in available family members.

## 3. Results

### 3.1. Patients

A total of 319 Chinese families with IRD were recruited for this study, including 24 autosomal dominant (AD), 33 autosomal recessive (AR), 11 X-linked, and 251 sporadic cases which included those whose inheritance pattern could not be determined. Of these patients, 220 patients were initially diagnosed with RP, 37 were diagnosed with cone-rod dystrophy, and 11 were initially diagnosed with Usher syndrome. These three diseases accounted for 84.0% of all IRD cases in this study. The remainder included Leber congenital amaurosis (LCA), congenital stationary night blindness (CSNB), Stargardt disease, and so on.

### 3.2. Pathogenic Mutations Identified

In targeted panel sequencing, pathogenic or likely pathogenic mutations were successfully identified in a total of 94 of 228 families, achieving a diagnostic rate of 41.2% (Table S1). With WES, 30 of 91 families were successfully diagnosed, achieving a diagnostic rate of 33.0% (Table S2). HEDEP resulted in a higher detection rate than WES examination. In addition, eight patients subjected to HEDEP and one patient subjected to WES were found to carry pathogenic mutations in the *RPGR* ORF15 region with Sanger sequencing (Table S3). Collectively, 132 of 319 families (41.4%) were genetically diagnosed.

**Figure 1:**
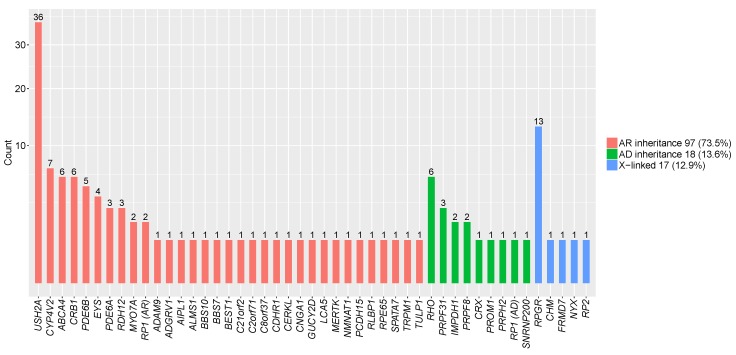
Genes with pathogenic or likely pathogenic mutations identified in 132 inherited retinal dystrophy (IRD) families which were genetically diagnosed. The percentage of inheritance patterns identified in this study. Prevalence of mutations in genes causing autosomal dominant (AD) IRD, autosomal recessive (AR) IRD and X-linked IRD.

Among these 132 families, 18 (13.6%) carried AD mutations, 97 (73.5%) carried AR mutations, and 17 (12.9%) were found to be X-linked (Figure 1). With six families harboring mutations in *RHO*, *RHO* is the most common disease-causing gene in Chinese patients with AD IRD (Figure 1). With 36 families (27.2%) harboring mutations in *USH2A*, *USH2A* is the most common disease-causing gene in Chinese patients with AR IRD (Figure 1). In families with mutations identified in X-linked genes, eight were sporadic with no family history. With 13 patients carrying mutations in *RPGR*, *RPGR* is the most common X-linked disease-causing gene (Figure 1). Accurate genetic diagnoses paved the way for genetic counseling and family planning for these patients. Among the 163 disease-causing variants identified in this study, 73 had been previously reported, and the other 90 were novel (Table S4).

### 3.3. Revision of the Initial Clinical Diagnosis in One Patient

After reevaluation, the clinical diagnosis for patient 2016090902 who carried the novel compound heterozygous mutations NM_024685.3:c.1063C>T:p.(Gln355Ter) and NM_024685.3: c.378G>A:p.(Trp126Ter) in *BBS10*, was revised to Bardet–Biedl syndrome. This patient was initially diagnosed with RP at the age of 8 years and was re-examined at the age of 12 years. At that time, other features of this syndrome such as obesity, hypogonadism, and polydactyly were identified (Figure S1). This result argues molecular diagnosis can play a significant role in accurate clinical diagnosis.

### 3.4. Observation of Mutations in ALMS1 Causing Non-Syndromic Retinal Dystrophy

A 10-year-old girl (patient 2016101713) was found to carry compound heterozygous mutations in *ALMS1* NM_015120.4:c.9154_9155delCT:p.(Cys3053SerfsTer9) and NM_015120.4:c.10825C>T: p.(Arg3609Ter) (Figure S2). The variant p.(Cys3053SerfsTer9) was novel, and the variant p.(Arg3609Ter) has been previously reported [23]. Mutations in *ALMS1* have been reported to cause Alstrom syndrome, but this patient did not show characteristic Alstrom syndromic features such as obesity, hearing loss, diabetes mellitus, and learning difficulties. This further supports a previous report which demonstrated mutations in *ALMS1* can cause non-syndromic retinal dystrophy [24,25].

### 3.5. Sequence Coverage and Bioinformatics Analysis

**Figure 2:**
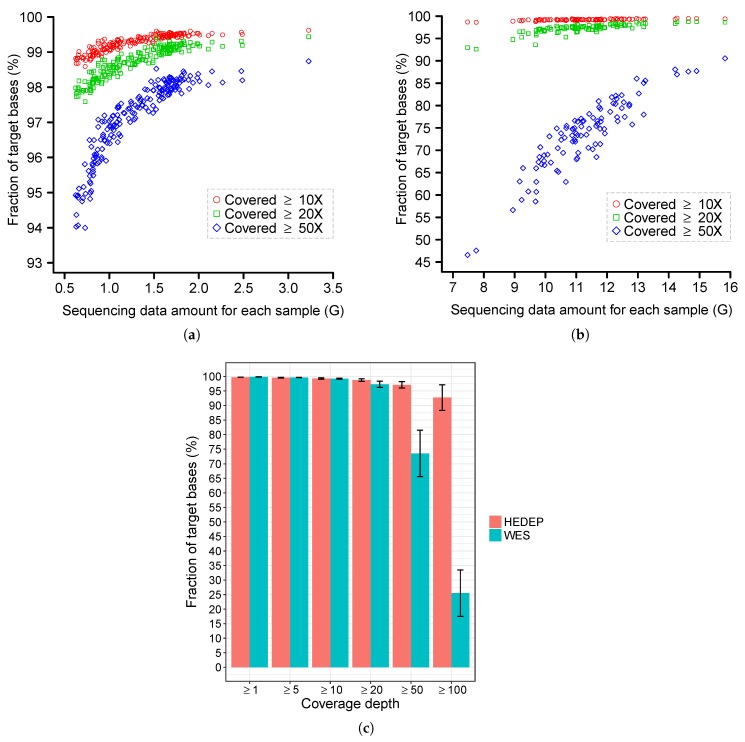
Coverage analysis for hereditary eye disease enrichment panel (HEDEP) and whole exome sequencing (WES). (**a**) The *x*-axis indicates the amount of clean data (after removing low quality reads from raw sequencing results) for each sample subjected to HEDEP. The *y*-axis indicates the fraction of target base pairs that had at least 10×, 20×, and 50× coverage for each sample, respectively. (**b**) The *x*-axis indicates the amount of clean data for each sample subjected to WES. The *y*-axis indicates the fraction of target base pairs (exonic regions for 441 hereditary eye disease genes) that had at least 10×, 20×, and 50× coverage for each sample, respectively. (**c**) Sequencing coverage comparison of HEDEP and WES. Bar plots show the fraction of target base pairs (exonic regions for 441 hereditary eye disease genes) that were covered to at least the depth indicated on the *x*-axis. Error bars indicate standard deviations.

A total of 228 IRD probands were subjected for HEDEP, which generated an average of 4.3 million 150 bp paired-end reads per sample (with an average depth of 331.8×). An average of 99.27%, 98.71%, and 97.11% of targeted bases had at least 10×, 20×, and 50× coverage, respectively. Sequencing depth and coverage analysis for each sample with HEDEP examination are shown in Figure 2. In comparison, the other 91 samples were processed for WES, which generated an average of 37.6 million reads per sample (with an average depth of 79.8×). An average of 98.92%, 96.20%, and 69.39% of bases had at least 10×, 20×, and 50× coverage, respectively. Focusing only on 441 hereditary eye disease genes, an average of 99.23%, 97.28%, and 73.58% of exonic regions of these genes had at least 10×, 20×, and 50× coverage, respectively. Sequencing depth and coverage analysis for each sample subjected to WES are shown in Figure 2. Overall, the samples subjected to HEDEP had better target region coverage than those subjected to WES. This is more obvious when comparing coverage with at least 50× (Figure 2).

**Figure 3:**
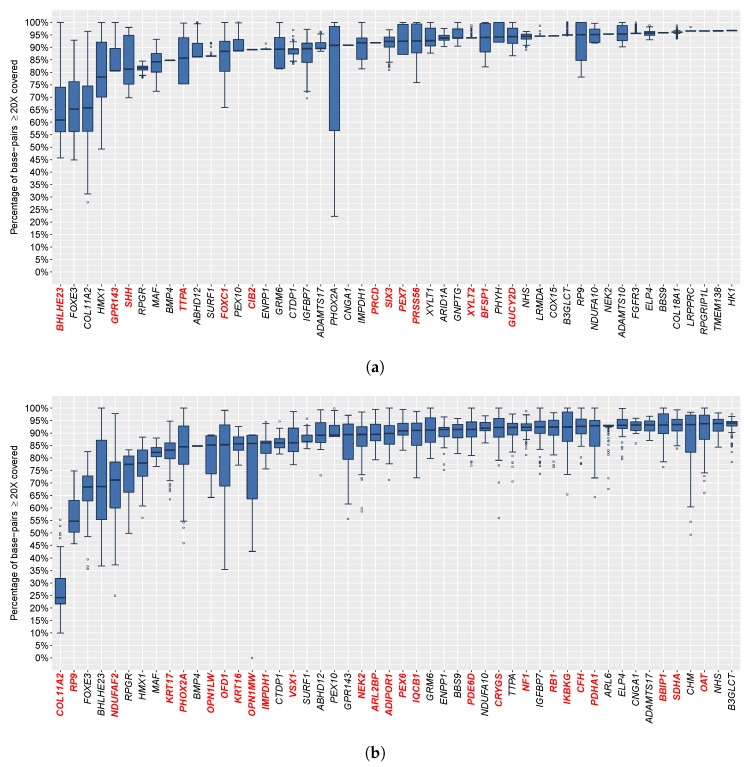
Overview of 50 genes with the lowest coverage. The boxplots depict the percent distribution of exonic regions with at least 20× coverage for the hereditary eye disease enrichment panel capture (**a**) and whole exome sequencing libraries (**b**). The poor coverage in a total of 27 genes was consistent for the two different capture methods. Some genes in one enrichment capture platform had coverage lower by more than 5% of the targeted regions than that in the other platform. These genes are highlighted in red and by bold font.

We next analyzed the sequencing coverage of 441 individual hereditary eye disease genes, including 291 genes related to IRD. In our data, 222 genes were 100% covered by at least 20× across all 228 samples subjected to HEDEP. In a total of 403 genes, at least 95% (averaged for 228 samples) of their exonic regions were covered by at least 20×. In contrast, only 43 genes were 100% covered by at least 20× across the 91 samples subjected to WES. In a total of 371 genes, at least 95% (averaged for 91 samples) of their exonic regions were covered by at least 20×. Fifty genes with lower coverage are shown in Figure 3 for HEDEP and WES, respectively. A total of 41 genes subjected to HEDEP had significantly better coverage (more than 5% greater coverage), including 26 genes which are highlighted in red and by bold font in Figure 3b. A total of 13 genes had significantly better coverage with the WES platform. These genes are highlighted in red and by bold font in Figure 3a.

## 4. Discussion

In this study, we identified 163 disease-causing mutations, of which 90 were novel. These data expand the IRD mutation spectrum and provide new targets for IRD diagnosis and treatment. The *RPGR* ORF15 region is highly repetitive and purine-rich, which are difficult to capture with NGS, and Sanger sequencing is an effective supplement for NGS for these conditions. As a result of family planning over the past thirty years in China, some patients are the only child in the family, making it impossible to determine the exact inheritance pattern and recurrence risk in offspring. However, with a genetic diagnosis, accurate genetic counseling can be provided and family planning can be offered [26,27].

Next generation sequencing offers a rapid and effective method for the molecular diagnosis of IRD. Both HEDEP and WES demonstrated a significantly high capture efficiency, but HEDEP had a higher diagnostic yield. There are several possible reasons for this. First, the samples subjected to HEDEP had better target region coverage than those subjected to WES. This indicates that the number of sufficiently covered coding sequences detected by HEDEP was much greater than those detected by WES, which result in improved accuracy of variant identification. Secondly, the difference in percentages of the different diseases in the two groups may influence the detection rates [28]. For example, Usher syndrome has a markedly higher diagnostic yield than RP, and predominantly recessive phenotypes tend to have higher mutation detection rates. Aside from above priority, HEDEP can also be designed to capture deep-intronic regions, which accounted for one third of cases in which only a single causative allele was identified [26]. Unfortunately, these deep-intronic regions were not included in this edition of HEDEP. Targeted sequencing also has shortcomings. Clinical panels are frequently out-of-date and laboratories must incur the never-ending incremental increases in cost of updating the contents of the panels as new genes are identified [29].

In contrast to targeted panel sequencing, WES is not limited to detection of known pathogenic genes [8,30,31,32,33,34]. When a new disease-causing gene is discovered, a bioinformatics re-analysis of the already available data is sufficient [26]. The ACMG recommends WES be considered in cases where the phenotype or family history are strongly suggestive of a genetic disorder but are non-specific, and for phenotypes where there is significant genetic heterogeneity, or in cases where more specific genetic tests have failed to yield a diagnosis [35]. However, WES has drawbacks, such as insufficient capture of the whole exome, as noted in this study. WES is also one-fold more expensive than HEDEP, and is therefore not recommend to be used as the primary tool for specific patients with IRD.

Both HEDEP and WES have limitations. First, neither can identify changes in the non-coding region [36]. Secondly, not all of the targeted exons have sufficient depth of coverage to accurately call variants, especially those with high GC content. Thirdly, small indels, large structural variants, CNVs, or duplications that may account for 9% of variants in Usher syndrome are not readily identified with NGS [37,38]. Whole-genome sequencing (WGS) would provide a solution to these problems. Since WGS can identify non-coding pathogenic variations, it is less sensitive to GC content and leads to coverage which is more uniform and complete than HEDEP or WES. This makes WGS more suitable for detection of CNV. However, the analysis of WGS data is complicated both due to the large amount of information, and to the challenges in determining whether specific genetic variants affect health [19]. Moreover, WGS is expensive and data storage is a significant burden that many patients and research institutions cannot afford. Thus, if a patient disease phenotype is specific, use of a gene panel is favored because of low sequencing costs, short turnaround time, and low rate of non-specific or incidental findings. If a disease phenotype is non-specific, WES is favored. However, if the panel or WES yield negative results, WGS may be considered as the most comprehensive secondary test [39].

In summary, NGS in conjunction with Sanger sequencing offers a rapid, effective, and accurate method for the molecular diagnosis of IRD, which helps provide a more accurate clinical diagnosis, and subsequently paves the way for family planning and gene-based treatment in affected patients.

## Supplementary Materials

The following are available online at http://www.mdpi.com/2073-4425/9/7/360/s1, Figure S1: Clinical phenotype and pathogenic mutations of *BBS10* identified in patient 2016090902; Figure S2: Clinical phenotype and pathogenic mutations of *ALMS1* identified in patient 2016101713; Table S1: Clinical data and pathogenic mutations are listed for 94 probands with hereditary eye disease enrichment panel; Table S2: Clinical data and pathogenic mutations are listed for 30 probands with whole exome sequencing; Table S3: Clinical data and pathogenic mutations are listed for 9 probands with *RPGR* ORF15 mutations; Table S4: Mutations identified in the present study.
