# Supplementary material for: Application of Whole Exome and Targeted Panel Sequencing in the Clinical Molecular Diagnosis of 319 Chinese Families with Inherited Retinal Dystrophy and Comparison Study
Source: Genes (Basel). 2018 Jul 19;9(7):360. doi: 10.3390/genes9070360 (PMC6071067; doi:10.3390/genes9070360)
Supplement: Supplementary file 1 [file genes-09-00360-s001.zip › FigureS1.pdf]

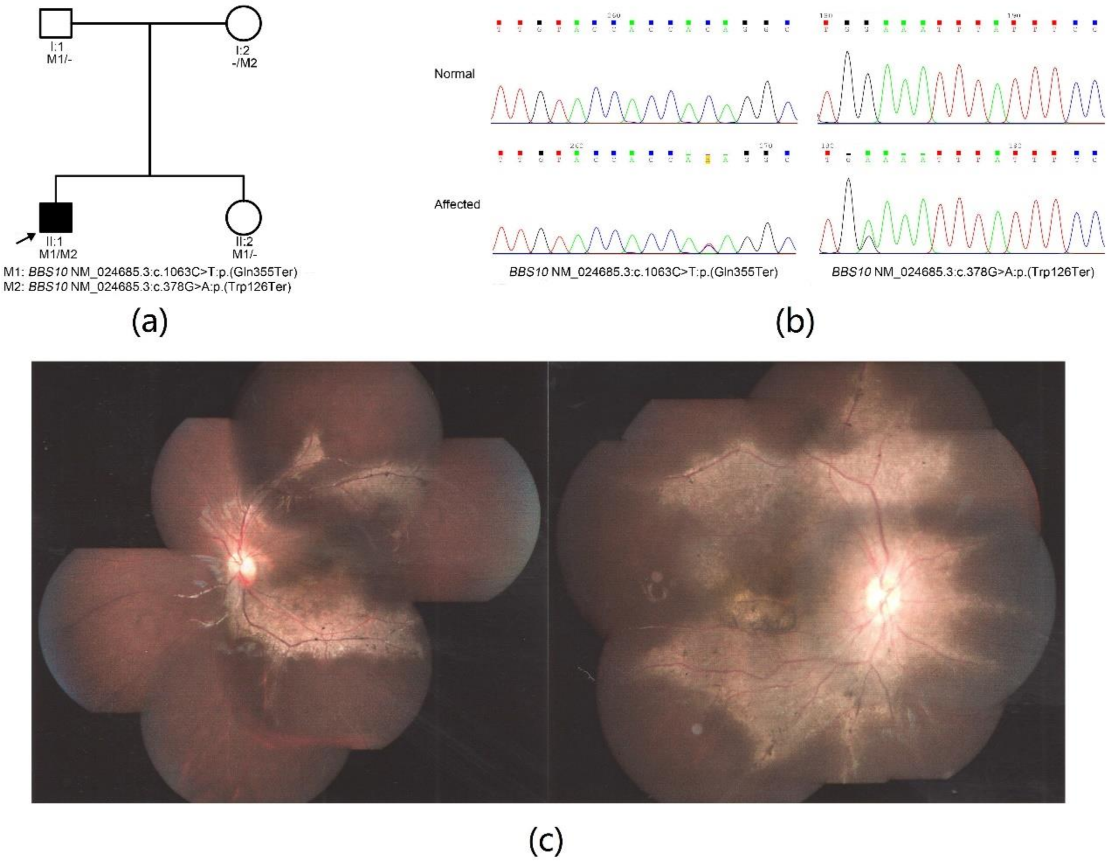

Figure S1. Clinical phenotype and pathogenic mutations of BBS10 identified in patient 2016090902. (a) The pedigree of the family. Arrow indicated the patient 2016090902; (b) Representative sequence chromatograms for the proband and his parents; (c) Fundus examinations of the proband showed widespread retinal atrophy in the posterior pole.
