# Supplementary material for: Application of Whole Exome and Targeted Panel Sequencing in the Clinical Molecular Diagnosis of 319 Chinese Families with Inherited Retinal Dystrophy and Comparison Study
Source: Genes (Basel). 2018 Jul 19;9(7):360. doi: 10.3390/genes9070360 (PMC6071067; doi:10.3390/genes9070360)
Supplement: Supplementary file 1 [file genes-09-00360-s001.zip › FigureS2.pdf]

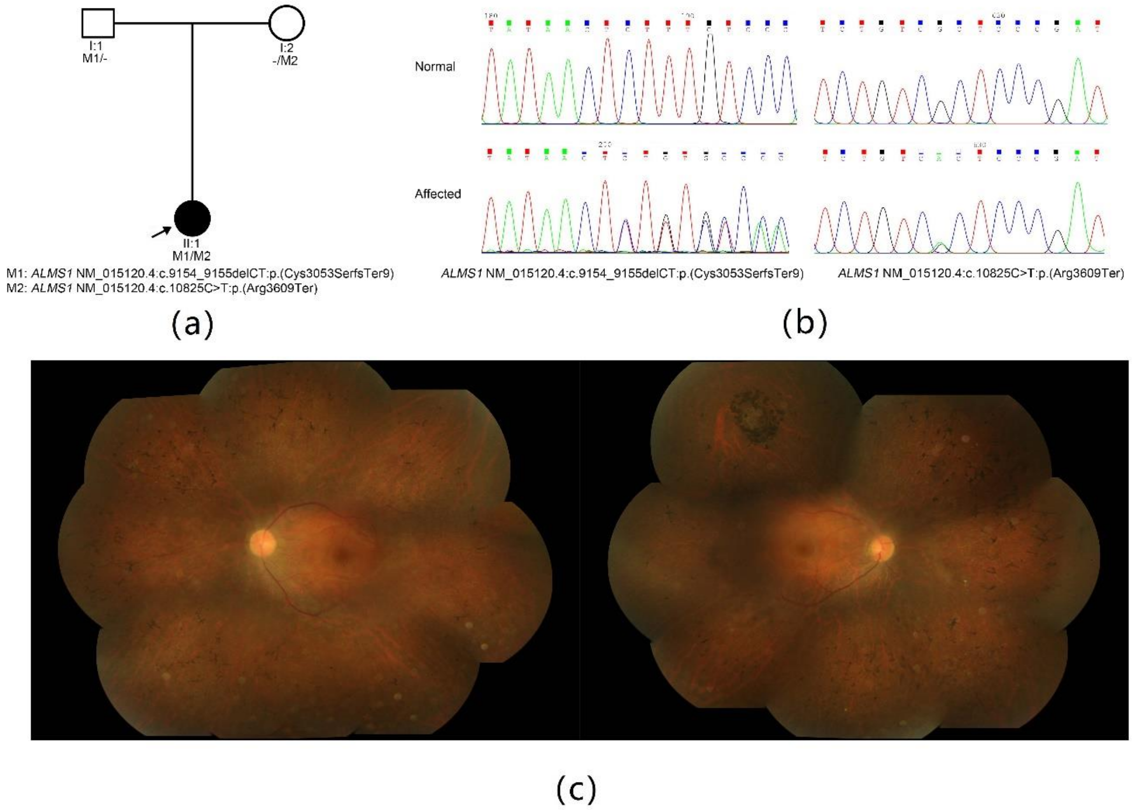

Figure S2. Clinical phenotype and pathogenic mutations of ALMS1 identified in patient 2016101713. (a) The pedigree of the family. Arrow indicated the patient 2016101713; (b) Representative sequence chromatograms for the proband and her parents; (c) The initial symptom of this patient was progressive central vision loss, her BCVA was found to be 0.1 and 0.15 in the right and left eye respectively. Fundus examination showed the symptoms of CORD, consisting of waxy pallor of the optic discs, retinal arteriolar attenuation, scattered bone-spicule pigmentation in the mid-peripheral retina, with macular was also involved.
